# Clay Minerals/TiO_2_ Composites—Characterization and Application in Photocatalytic Degradation of Water Pollutants

**DOI:** 10.3390/molecules29204852

**Published:** 2024-10-13

**Authors:** Bogna D. Napruszewska, Dorota Duraczyńska, Joanna Kryściak-Czerwenka, Paweł Nowak, Ewa M. Serwicka

**Affiliations:** Jerzy Haber Institute of Catalysis and Surface Chemistry, Polish Academy of Sciences, Niezapominajek 8, 30-239 Krakow, Poland; ncnaprus@cyf-kr.edu.pl (B.D.N.); dorota.duraczynska@ikifp.edu.pl (D.D.); joanna.krysciak-czerwenka@ikifp.edu.pl (J.K.-C.);

**Keywords:** TiO_2_, clay, montmorillonite, laponite, composite, inverse microemulsion, mesoporosity, photocatalysis, methyl orange, humic acid

## Abstract

TiO_2_ used for photocatalytic water purification is most active in the form of nanoparticles (NP), but their use is fraught with difficulties in separation from solution or/and a tendency to agglomerate. The novel materials designed in this work circumvent these problems by immobilizing TiO_2_ NPs on the surface of exfoliated clay minerals. A series of TiO_2_/clay mineral composites were obtained using five different clay components: the Na-, CTA-, or H-form of montmorillonite (Mt) and Na- or CTA-form of laponite (Lap). The TiO_2_ component was prepared using the inverse microemulsion method. The composites were characterized with X-ray diffraction, scanning/transmission electron microscopy/energy dispersive X-ray spectroscopy, FTIR spectroscopy, thermal analysis, and N_2_ adsorption–desorption isotherms. It was shown that upon composite synthesis, the Mt interlayer became filled by a mixture of CTA^+^ and hydronium ions, regardless of the nature of the parent clay, while the structure of Lap underwent partial destruction. The composites displayed high specific surface area and uniform mesoporosity determined by the size of the TiO_2_ nanoparticles. The best textural parameters were shown by composites containing clay components whose structure was partially destroyed; for instance, Ti/CTA-Lap had a specific surface area of 420 m^2^g^−1^ and a pore volume of 0.653 cm^3^g^−1^. The materials were tested in the photodegradation of methyl orange and humic acid upon UV irradiation. The photocatalytic activity could be correlated with the development of textural properties. In both reactions, the performance of the most photoactive composites surpassed that of the reference commercial P25 titania.

## 1. Introduction

Since the pioneering work of Fujishima and Honda [1], describing the phenomenon of water splitting into its constituent elements upon illumination of a TiO_2_ photoelectrode, photocatalysis has been seen as a cost-effective, clean and safe technology with great potential for the future [2,3]. Titania continues to be the most widely investigated photocatalyst due to its exceptional photoactivity, high chemical and thermal stability, non-toxicity, environmental friendliness, and low cost [4,5]. TiO_2_ has proven particularly effective in the form of nanoparticles (NPs), and the design of photocatalysts based on TiO_2_ NPs is now a rapidly expanding research area [6,7,8]. The advantages of using NPs include a high surface-to-volume ratio, high density of coordinatively unsaturated sites, modified electron structure, and altered adsorption and/or acid–base properties of the surface [9]. Manipulating nanoparticles, however, is fraught with many challenges, including the difficulties of their separation and recovery from the reaction medium, a tendency to form clusters, or a propensity to sinter when heated [10,11]. A possible solution to these problems can be the immobilization of nanoparticles on appropriate supports. This approach has often been applied to TiO_2_ NPs, and both inorganic and organic carriers have been used, including silica, zeolites, glass, clay minerals, carbon materials, or organic polymers [12,13,14,15].

Among different potential carriers of titania nanoparticles, clay minerals, especially those from the group of smectites, are of particular interest. Smectites are layered silicates that exhibit a unique combination of favorable properties; namely, they are widely distributed in nature, non-toxic, and inexpensive, and their lamellar lattice elements can be easily manipulated and used as ready-made building blocks for the synthesis of advanced nanomaterials [16,17,18]. Composites of clay minerals and titania have been known for almost 40 years, i.e., since Sterte [19] described the synthesis of Ti-pillared montmorillonite. Today, the attractiveness of TiO_2_/clay systems is determined by their potential in photocatalytic applications [14,20,21,22,23,24,25]. The clay mineral, by far the most commonly used in the engineering of composite structures, is montmorillonite. The mineral consists of layers built of two tetrahedral Si-based sheets sandwiching an octahedral Al-based sheet. Partial isomorphous substitution of Al^3+^ by Mg^2+^ in the octahedral sheet generates a negative charge on the clay layers, which is balanced by the presence of cations in the interlayer space. This opens a facile route for the use of montmorillonite in materials engineering. The naturally occurring simple interlayer cations, such as Ca^2+^ or Na^+^, can be readily exchanged for more complex positively charged species while maintaining parallel stacking of the layers, yielding pillared clays. On the other hand, due to the swelling ability, the layered smectite lattice may exfoliate and form disordered assemblies with TiO_2_ nanoparticles, creating a structure referred to as a house of cards. The TiO_2_ nanoparticles to be supported on clay carriers are usually generated by the in situ or ex situ hydrolysis of inorganic or organic Ti compounds, most commonly TiCl_4_ or Ti alkoxides.

We have recently proposed a new strategy for the preparation of hybrid structures combining TiO_2_ and montmorillonite (sodium or cetyl trimethylammonium (CTA) form) based on the synthesis of titania NPs in water-in-oil (w/o) microemulsions and mixing the microemulsion with an appropriately dispersed clay mineral component [26]. The new materials had a high specific surface area and could accommodate large amounts of TiO_2_ NPs evenly dispersed in a uniformly mesoporous structure. Because of these characteristics, composites prepared according to the designed procedure appear attractive as potential photocatalysts.

Accordingly, in the present work, we used this approach to prepare a series of five composites of clay minerals with titania to study their photocatalytic properties. In addition to Na- and CTA- montmorillonite, three new clay supports were used, as yet untested, in combination with TiO_2_ NPs prepared by inverse micellar route, namely acid-activated H-montmorillonite, Na-laponite, and CTA-laponite. Laponite is a synthetic hectorite, a clay mineral whose structure is similar to montmorillonite in terms of sheet arrangement and the presence of interlayer cations, but the octahedral sheet is Mg-based, and the layer charge results from the replacement of some Mg^2+^ by Li^+^. The prepared composites were subjected to a thorough physicochemical characterization and tested in the photocatalytic purification of water from organic contaminants. With the growing scarcity of clean water [27], the effective treatment of water pollution is a task of paramount importance, and TiO_2_-based photocatalysis is one of the most promising strategies for solving this problem [28,29,30]. The substances selected for photodegradation tests were methyl orange and humic acid. Methyl orange is a model compound for refractory dye pollutants released into wastewater from many sources, including textile, leather, printing, paper, food, chemical, and paint industries [31]. Humic acids, ever present in soils and water bodies, are macromolecules of high structural complexity formed in nature as a result of the long-term decomposition of biomass residues. These compounds are considered harmful contaminants of water resources used to produce drinking water due to the formation of highly toxic, carcinogenic, and mutagenic disinfection byproducts [32].

The photocatalytic performance of the synthesized composites was discussed in terms of its dependence on the physicochemical properties of the materials and compared with P25, a commercial titanium nanopowder with high photoactivity, widely used as a benchmark in TiO_2_-related research [33].

## 2. Results and Discussion

### 2.1. Chemical Composition

The clay–TiO_2_ composites were obtained using five different clay mineral components. Three of them were modifications of montmorillonite (Na-Mt, H-Mt, CTA-Mt), and two of laponite (Na-Lap, CTA-Lap). The chemical composition of investigated materials, based on EDX analysis, is presented in Table 1. The content of TiO_2_ in montmorillonite-based composites is about 60%, i.e., close to the intended titania loading. Laponite-derived composites contain more TiO_2_. The higher content of TiO_2_ in Ti/Na-Lap and Ti/CTA-Lap is attributed to the partial dissolution of laponite during composite synthesis. It is known that a reaction medium with acidic pH can destroy laponite structure [34]. In composite synthesis, the acidic environment is generated by the aqueous cores of Ti-bearing inverse micelles. This may damage the laponite lattice, although the final neutralization step with ammonia can lead to its reconstruction and/or precipitation of another form of magnesium silicate. Chemical analysis evidences the retention of clay-forming elements in the laponite-derived composites, but partial loss of Mg with respect to Si is observed, pointing to the occurrence of the preferential dissolution of magnesium. Noteworthy, the composites prepared from the sodium form of montmorillonite or laponite are free of Na, indicating that full cation exchange has occurred during synthesis.

### 2.2. X-ray Diffraction Analysis

**Figure 1:**
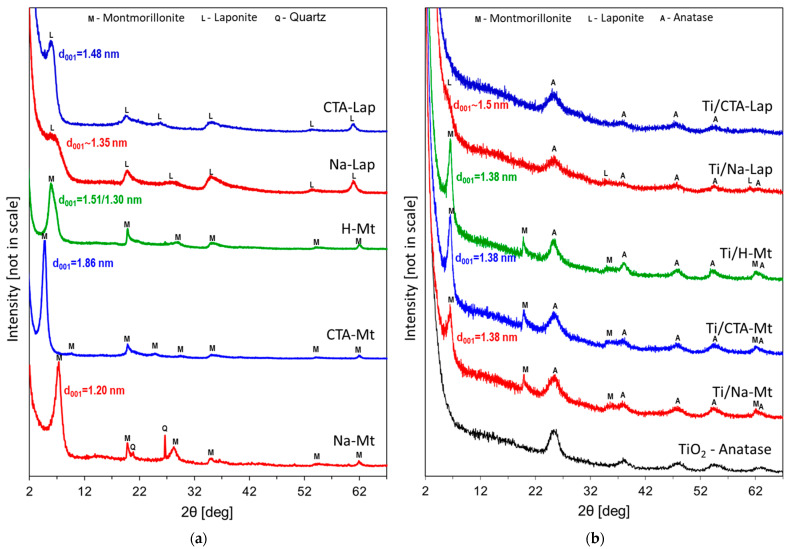
XRD patterns of (**a**) clay minerals used as supports for TiO_2_ nanoparticles; (**b**) composites of TiO_2_ and clay minerals.

The XRD patterns of clay supports used for the composite synthesis are shown in Figure 1a. The maximum basal (001) reflection of the sodium form of montmorillonite, Na-Mt, appears at d_001_ = 1.20 nm, as expected for the monohydrated Na–smectite layer [35]. In addition, Na-Mt contains traces of quartz impurity. CTA-Mt organoclay, formed by the exchange of interlayer sodium cations with CTA cations, is characterized by the interlayer distance d_001_ = 1.86 nm. Such a value points to the bilayer conformation adopted by organic aliphatic chains within the interlayer [36]. The basal reflection in CTA-Mt shows an increased intensity relative to other montmorillonite reflections, which indicates a greater number of well-oriented stacks of clay platelets. The interlayer distance in the proton-exchanged montmorillonite is known to depend strongly on the degree of hydration. The H-Mt sample shows a basal reflection with a maximum at 1.51 nm, characteristic of the presence of two interlamellar water layers surrounding hydronium ions, while a shoulder around 1.30 nm indicates some degree of interstratification involving areas with one and two water layers [37]. Interstratification and, possibly, the poorer orientation of acid-damaged clay platelets are responsible for the lower relative intensity of basal reflection in the H-Mt sample. Na-Lap displays a broad maximum around d_001_ = 1.35 nm while the d_001_ of organoderivative CTA-Lap equals 1.48 nm, the value typical of a monolayer arrangement of alkyl chains. Both results are in agreement with previous studies [38]. Similarly, as in the case of montmorillonite, the higher relative intensity of basal reflection in organoclay indicates a better stacking of individual platelets than in the Na-Lap sample.

Figure 1b shows the XRD patterns of the synthesized titania–clay composites. In the case of composites based on montmorillonite, reflections due to both composite components are visible. The titania component displays the features of nanocrystalline anatase. This is clearly visible by comparison with the XRD pattern of reference TiO_2_ nanoparticles with anatase structure, obtained ex situ by the inverse micellar route. As to the clay components, the d_001_ spacing assumes the same value of 1.38 nm, characteristic of a monolayer arrangement of alkylammonium cations in the interlayer, irrespective of the nature of the parent clay, be it Na-Mt, CTA-Mt, or H-Mt. For CTA-Mt and H-Mt, it signifies a decrease in the interplanar spacing, but in the case of Na-Mt, an increase is observed. The result indicates that in the conditions of composite formation, the interlayer space in all clay components tends to acquire a similar filling. Indeed, during the mixing of composite components, the reaction medium contains a high concentration of hydronium ions stemming from the acidic aqueous cores of Ti inverse micelles and of CTA^+^ cations from the surfactant required for the stabilization of aqueous nanodroplets. Thus, the observed effects can be attributed to cation exchange processes that lead to the occupation of the interlayer space of all clay components by a mixture of hydronium and CTA^+^ cations. In the case of CTA-Mt, the partial exchange of interlayer CTA^+^ cations for hydronium ions reduces the content of CTA^+^ in the interlayer and allows monolayer packing of organocations, which is the reason for the shift of d_001_ from 1.86 nm to 1.38 nm. The change in the interlayer distance in the H-Mt component from 1.51 nm to 1.38 nm is due to the replacement of some H_3_O^+^ cations by CTA^+^ cations, which assume flat, single-layer conformation. The resulting hydrophobization of the interlayer lowers the amount of water coordinated to the remaining hydronium ions so that the interlayer distance is determined by the thickness of the organic tail of CTA^+^. In the case of the Na-Mt component, the increase in d_001_ from 1.20 nm to 1.38 nm can be explained by the replacement of interlayer Na^+^ by a mixture of CTA^+^ and hydronium ions. This conclusion is supported by the results of EDX analysis, which found no sodium in the Ti/Na-Mt composite (Table 1). The Ti/Na-Lap sample shows very weak XRD features of the clay component with d_001_ approximately equal to ca. 1.5 nm, which would suggest at least partial conversion to CTA^+^-exchanged laponite. Similarly, as in the case of Ti/Na-Mt, EDX analysis shows that the Ti/Na-Lap composite is free of sodium. No reflections attributable to the clay mineral are observed in the XRD pattern of the Ti/CTA-Lap composite. This may be due to the delamination of the clay component or to the destruction of its lattice upon the action of the acid environment. This aspect is discussed in more detail in the next section, which describes the results of the FTIR analysis. Anatase reflections are visible in both Ti/Na-Lap and Ti/CTA-Lap, although they are broader than in composites derived from montmorillonite. Indeed, the crystal sizes of TiO_2_ nanoparticles in the laponite-based samples, estimated from line broadening using the Scherrer equation, are smaller than those calculated for composites prepared from montmorillonite components (Table 1).

### 2.3. FTIR Spectroscopy

To assess whether the laponite component retained its structural identity after contact with an acidic environment, Ti/Na-Lap and Ti/CTA-Lap samples were subjected to FTIR analysis, and their spectra were compared with those of laponite carriers and nanocrystalline anatase.

**Figure 2:**
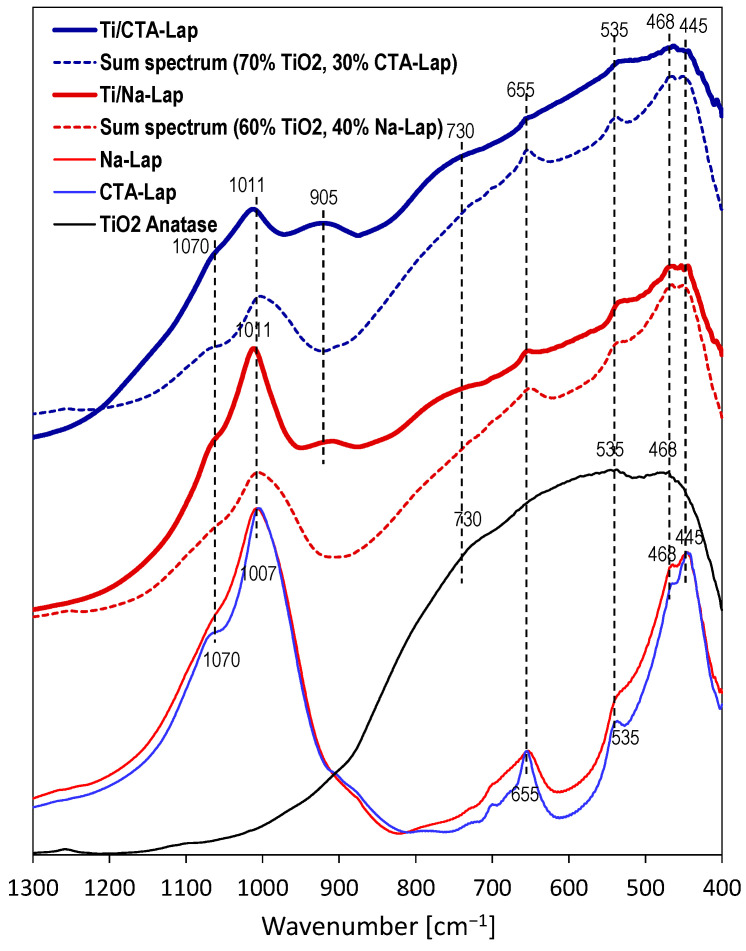
FTIR spectra of laponite-based composites, clay supports, reference nanocrystalline TiO_2,_ and sum spectra of individual components.

Figure 2 shows infrared spectra in the 1300–400 cm^−1^ range, where skeleton modes appear. The spectra of Na-Lap and CTA-Lap components are very similar, and their most prominent bands are marked. The strong maximum at 1007 cm^−1^ is due to Si-O stretching vibrations in the tetrahedral sheet, while the shoulder at 1070 cm^−1^ stems from Si-O bond vibrations with orientation perpendicular to the basal plane of the oxygens. The maximum at 655 cm^−1^ is due to OH bending vibrations in Mg_3_OH units in the octahedral sheet. The 535 cm^−1^ band corresponds to perpendicular Mg-O vibrations, while the two peaks at 468 and 445 cm^−1^ are due to overlapping Si-O-Mg and Si-O-Si bending vibrations [39]. Anatase nanoparticles obtained by the inverse micellar method show a broad band with weak maxima at 438 and 535 cm^−1^ and a shoulder at 720 cm^−1^, all attributed to vibrations in the anatase structure [40]. Figure 2 shows that the spectra of the composites exhibit features of both components. This is best illustrated by the presented sum spectra of TiO_2_ and laponite carriers, which show clear similarity to the spectra of composites, even though they are very approximate in nature. It should also be noted that in order to obtain sum spectra with a comparable degree of similarity to those of the actual composites, a smaller proportion of the laponite spectrum was required for Ti/CTA-Lap. Moreover, the laponite features in the Ti/CTA-Lap spectrum are more broadened than in the Ti/Na-Lap spectrum, suggesting a higher degree of structural damage in the latter. The only band that is present in the composites and absent in the simple sums of the component spectra is the band at 905 cm^−1^, more prominent in Ti/CTA-Lap. In silicates, this can be attributed to Q^2^ sites, i.e., tetrahedral SiO_4_ species with two bridging oxygens [41]. The result can be taken as evidence of some depolymerization of laponite silicate sheets, with the possible formation of a new amorphous magnesium silicate phase.

In summary, the above data, together with the results of XRD and chemical analysis, indicate that although the laponite components have been partially destroyed/dissolved, at least part of the clay component present in the composites has retained the structural identity of laponite, especially in Ti/Na-Lap, while the other part likely forms an amorphous chain silicate phase.

### 2.4. Electron Microscopy Analysis

**Figure 3:**
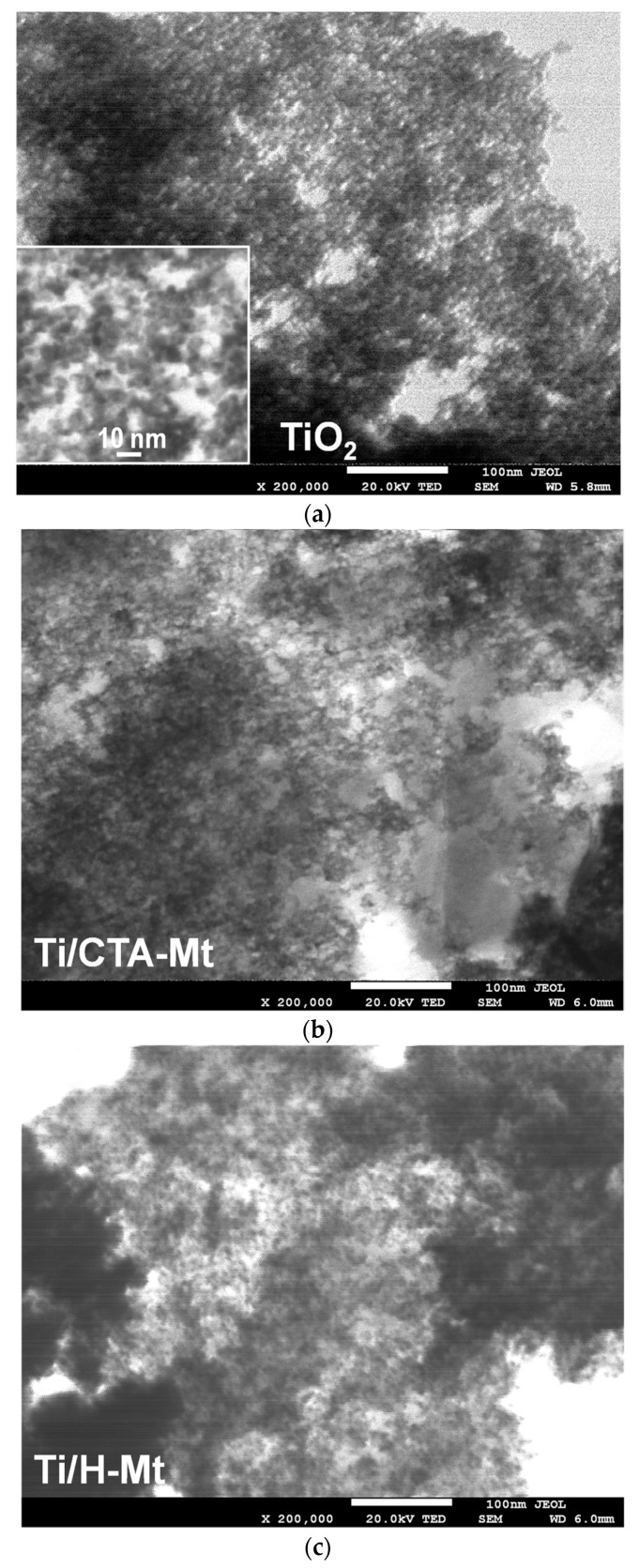
TEM images of (**a**) nanoparticles present in hydrolyzed Ti-containing inverse microemulsion; (**b**) Ti/CTA-Mt composite; (**c**) Ti//H-Mt composite. Sample suspensions deposited on 200 mesh copper grids covered with carbon film. Magnification ×200,000.

Electron microscopy analysis was used to evaluate the degree of titania dispersion on clay supports. This aspect was of particular interest due to the high TiO_2_ content in the synthesized composites. TEM image of Ti-containing inverse microemulsion, hydrolyzed without contact with clay component, is shown in Figure 3a. It can be seen that the microemulsion contains very fine rounded nanoparticles about 4–6 nm in diameter. Figure 3b,c show, as examples, TEM micrographs of Ti/CTA-Mt and Ti/H-Mt. TiO_2_ appears in the images as darker spots, while the clay mineral component is visible as lighter-colored areas. The micrographs reveal that the TiO_2_ component present in the composites looks similar to nanoparticles precipitated in microemulsion, which had no contact with clay support. In Ti/H-Mt, the TiO_2_ nanoparticles are quite homogeneously dispersed (Figure 3c), while in the Ti/CTA-Mt composite, the TiO_2_ coverage of the clay surface looks somewhat less uniform (Figure 3b).

It should be noted that particles formed in aqueous cores of inverse micelles, such as those present in composites, cannot be separated and preserved ex situ. The particles retain their size only when they are immobilized on the exfoliated clay mineral. As described in our recent work [40], after the separation of TiO_2_ formed in inverse microemulsions, their agglomeration and recrystallization occur during washing and drying, resulting in nanoparticles of larger size, different morphology, and different phase composition.

**Figure 4:**
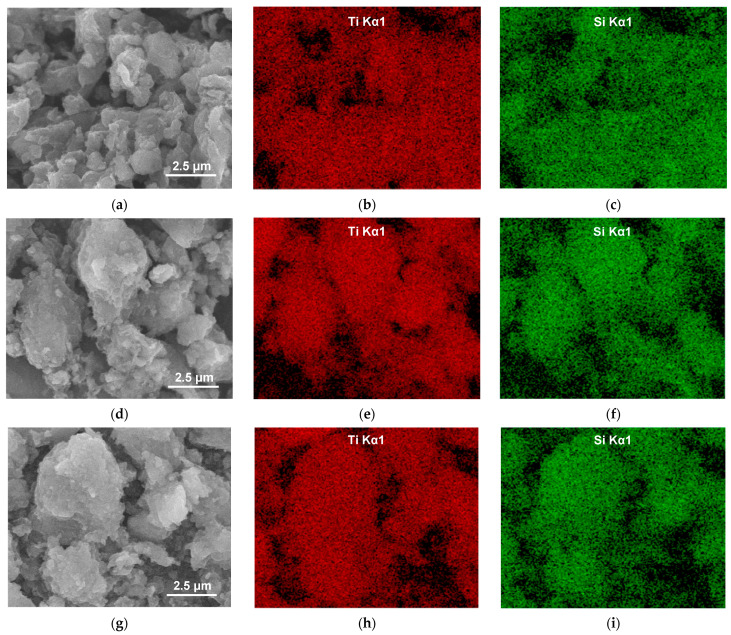

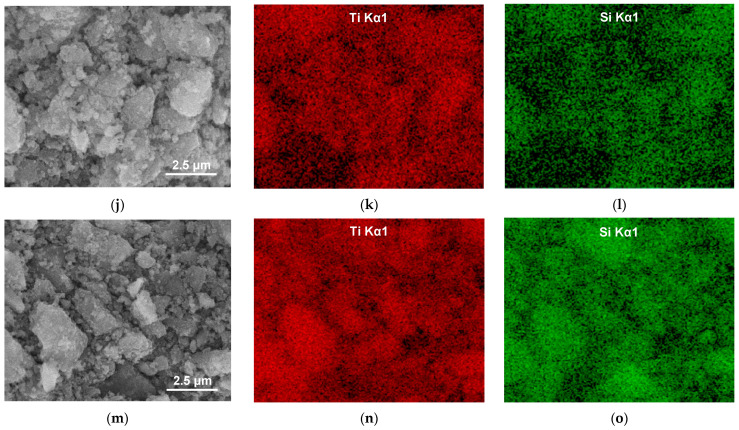
SEM/EDX compositional analysis of selected areas of synthesized composites: (**a**) SEM image of Ti/CTA-Mt; (**b**) EDX mapping of Ti in Ti/CTA-Mt; (**c**) EDX mapping of Si in Ti/CTA-Mt; (**d**) SEM image of Ti/Na-Mt; (**e**) EDX mapping of Ti in Ti/Na-Mt; (**f**) EDX mapping of Si in Ti/Na-Mt; (**g**) SEM image of Ti/H-Mt; (**h**) EDX mapping of Ti in Ti/H-Mt; (**i**) EDX mapping of Si in Ti/H-Mt; (**j**) SEM image of Ti/CTA-Lap; (**k**) EDX mapping of Ti in Ti/CTA-Lap; (**l**) EDX mapping of Si in Ti/CTA-Lap; (**m**) SEM image of Ti/Na-Lap; (**n**) EDX mapping of Ti in Ti/Na-Lap; (**o**) EDX mapping of Si in Ti/Na-Lap.

To better assess the distribution of TiO_2_ in the samples, SEM/EDX mapping was performed for all the composites studied. Figure 4 allows a comparison of the SEM image of the studied sample with the EDX mapping of titanium (reflecting the distribution of the photoactive component) and silicon (the key structure-forming element of the clay mineral support). It can be seen that for a given composite, the mappings of Ti and Si approximately mirror each other and reflect the texture/morphology of clay particles visible in SEM images. Thus, EDX mapping shows that in all samples, titanium is dispersed throughout the analyzed material, indicating uniform mixing of TiO_2_ nanoparticles with the clay component.

### 2.5. Thermal Analysis

**Figure 5:**
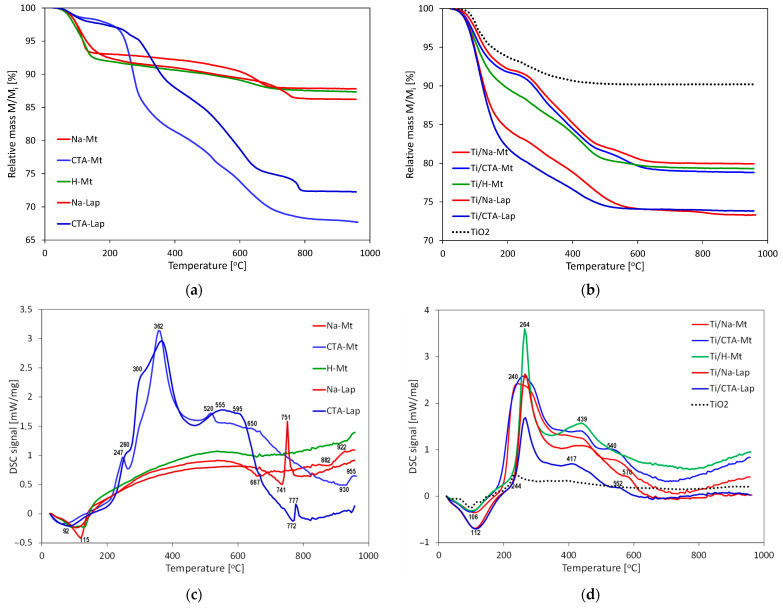
(**a**) TG traces of clay components; (**b**) TG traces of Ti/clay composites and of reference TiO_2_; (**c**) DSC traces of clay components; (**d**) DSC traces of Ti/clay composites and of reference TiO_2_.

TG/DSC analysis conducted for the investigated materials provided further evidence of a change in the interlayer composition of clay components during composite formation. Figure 5a,b show, respectively, the TG traces of parent clays and of composites, while the corresponding DSC profiles are displayed underneath, in Figure 5c,d. In general, in the presented TG traces, three temperature ranges can be distinguished, in which different decomposition processes occur. In the first stage, below 180 °C, dehydration of clay and/or TiO_2_ nanoparticles occurs due to the desorption of adsorbed water and/or loss of water present in the clay interlayer. The effects are accompanied by the endothermic maxima around 90–120 °C. The second stage is limited on the high-temperature side by a plateau. In this temperature range, the endothermic dehydroxylation of clay layers and exothermic oxidative decomposition of the surfactant molecules, present as interlayer CTA^+^ cations or adsorbed at the composite surface, occur. In the third stage of thermal evolution, the mass of the samples remains constant, and the exothermic effects that appear in this range, sometimes preceded by weaker endothermic minima, are due, respectively, to the crystallization of new phases and final breakdown of clay lattice.

Both in Figure 5a,b two groups of TG traces of significantly different total weight loss can be distinguished. However, the differentiating factors are not the same. In the case of pure clay components, it is the presence of organic cations in the interlayer and their combustion that are responsible for the higher weight loss in CTA-Mt and CTA-Lap. On the other hand, the TG profiles of composites are grouped according to the type of clay matrix, with those derived from laponite showing greater weight loss, occurring mainly on account of dehydration.

The TG curves of parent clays (Figure 5a) show that in CTA-Mt and CTA-Lap, which, due to the organic cations in the interlayer, are relatively hydrophobic, the water loss related to dehydration is much lesser than in the clays with inorganic interlayer cations. The combustion of the organic cations is a complex process that occurs in several overlapping steps accompanied by strong exothermic maxima. In Na-Mt, the final mass loss due to the dehydroxylation of montmorillonite layers occurs at 667 °C, as indicated by the associated exothermic effect. In CTA-Mt, it cannot be distinguished from the principal mass loss caused by combustion of the organic matter, and in H-Mt, the thermal effects due to structural transformations remain unresolved. In Na-Lap, the dehydroxylation is completed at 741 °C, while in CTA-Lap at 772 °C. Recrystallization of decomposed Na-Mt and CTA-Lap occurs at 922 and 955 °C, while that of Na-Lap and CTA-Lap at 751 and 777 °C, respectively. The data show that both organoclays are thermally more stable than their inorganic counterparts, which is in accordance with previous reports [42,43].

The total weight losses of the composites derived from montmorillonite are similar, and the same is true for two laponite-based samples, for which the overall effect is stronger. However, the distribution of weight loss between the first and the second stage of decomposition shows some differences, especially evident in the case of Ti/CTA-Lap and Ti/Na-Lap. In Ti/CTA-Lap, the dehydration effect is much more pronounced than in Ti/Na-Lap, while the reverse is true of the second stage weight loss, involving the combustion of the organic matter (Figure 5b). The DSC profiles of all composites are dominated by strong exothermic effects due to the oxidation of organic species (Figure 5d). For comparison, Figure 5b,d also show TG/DSC data for nanocrystalline TiO_2_. Although a small exothermic effect can be found in the DSC profile at 244 °C, it is clear that the thermal evolution of the composites in the second temperature range is related primarily to the properties of the clay component. These observations confirm that during the synthesis of the composites, the interlayer filling of clay components other than CTA-Mt and CTA-Lap tends to depart from that of the parent clays by acquiring a certain degree of loading with organic cations.

### 2.6. Textural Analysis

**Figure 6:**
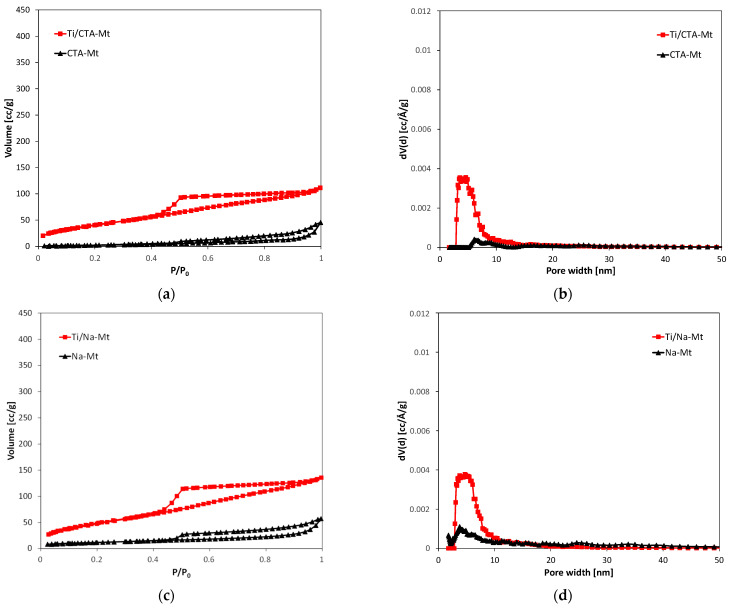

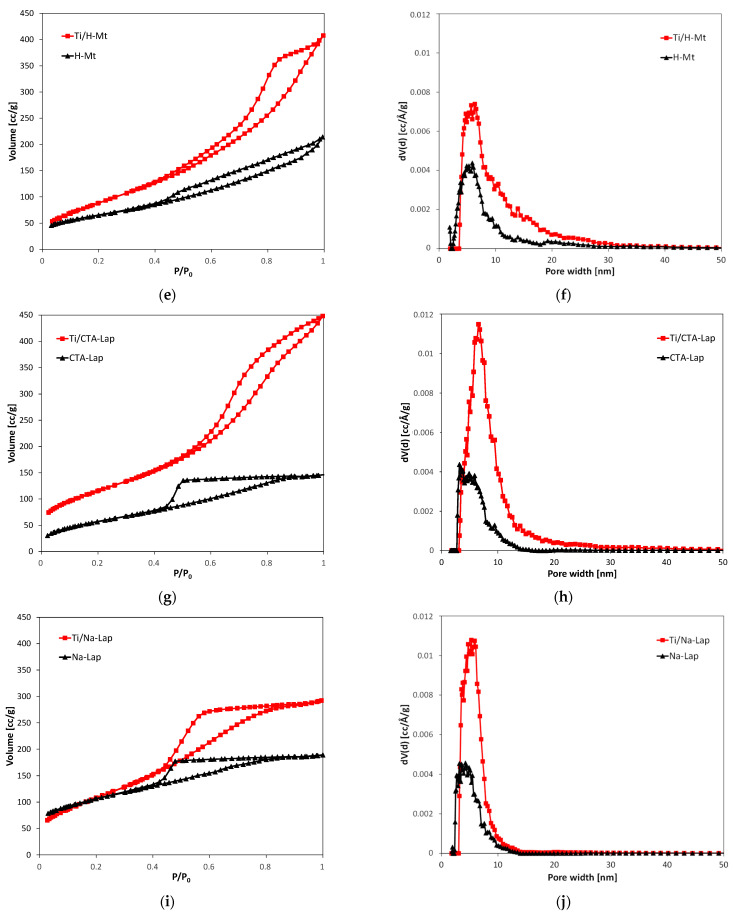
N_2_ adsorption/desorption isotherms at −196 °C of (**a**) CTA-Mt and Ti/CTA-Mt; (**c**) Na-Mt and Ti/Na-Mt; (**e**) H-Mt and Ti/H-Mt; (**g**) CTA-Lap and Ti/CTA-Lap; (**i**) Na-Lap and Ti/Na-Lap. Differential pore size distribution of (**b**) CTA-Mt and Ti/CTA-Mt; (**d**) Na-Mt and Ti/Na-Mt; (**f**) H-Mt and Ti/H-Mt; (**h**) CTA-Lap and Ti/CTA-Lap; (**j**) Na-Lap and Ti/Na-Lap.

The nitrogen adsorption/desorption isotherms and corresponding pore size distribution curves of all composites and clays from which they were obtained are shown in Figure 6, and the calculated textural parameters are presented in Table 1.

Na-Lap and CTA-Lap clays exhibit isotherms that level off at high relative pressures, described as type IVa, according to the IUPAC classification [44]. Hysteresis loops are of the H2a type and are characterized by a steep desorption branch and a flat plateau. H2a loops occur in materials with complex pore structures, consisting of interconnected pore networks of different sizes and shapes. The Ti/Na-Lap isotherm has a similar shape, but the Ti/CTA-Lap isotherm has a mixed character and is closer to type II with a less steep H2b hysteresis loop. All isotherms of parent montmorillonite clays and their composites can be classified as type II with H3-type hysteresis loops, known to be given by non-rigid aggregates of plate-like particles.

It is clear that the formation of composites had a profound effect on the texture of materials. In all cases, an increase in the specific surface area and nitrogen sorption capacity is observed. The effect is attributed to the formation of a new pore network due to the mixing of exfoliated clay minerals and TiO_2_ nanoparticles. Not surprisingly, the parent clays with the highest specific surface area, i.e., Na-Lap, CTA-Lap, and H-Mt, yield composites with the best-developed texture. It should be noted, however, that the particularly pronounced texture enhancement of the CTA-Lap-based composite (doubling of specific surface area and tripling of total pore volume) is likely to be due in part to the formation of an amorphous silicate phase, the presence of which has been suggested on the basis of infrared spectra. The presence of relatively narrow maxima in the pore size distribution curves shows that the newly generated porosity is quite uniform and within the range of small mesopores. The dominant pore sizes are around 5–6 nm, which is close to the size of TiO_2_ particles determined from TEM images (4–6 nm). Thus, it may be presumed that it is the presence of monodisperse TiO_2_ nanoparticles formed within inverse micelles that has the greatest impact on shaping the mesoporosity of the composites.

### 2.7. Photocatalytic Experiments

The photocatalytic properties of the synthesized composites were evaluated by testing their activity in the photodegradation of methyl orange and humic acid, and the results were referenced to the performance of commercial titania P25. The investigations began by measuring the decomposition of both MO and HA in the absence of a catalyst. No measurable decomposition of either HA or MO was observed, which was attributed to the application of low-energy UV radiation. The energy of the radiation source (365 nm, 3.40 eV) was only ~0.2 eV above the bandgap energy of TiO_2_ (anatase). It should also be noted that the use of low-energy radiation allows better utilization of the energy of the radiation source since lower quantum energy means a higher number of quanta generated at a given power of the radiation source. In the dark, the composites adsorbed a negligible amount of HA and about 15% of the total amount of OM present in the solution. After equilibration in the OM solution the catalysts, which were originally white (Lap-based) or ivory (Mt-based), acquired an orange color, which paled to yellowish after photocatalytic experiments. Exposure of the catalysts to the HA solution in the dark or after illumination did not cause any significant change in their color.

**Figure 7:**
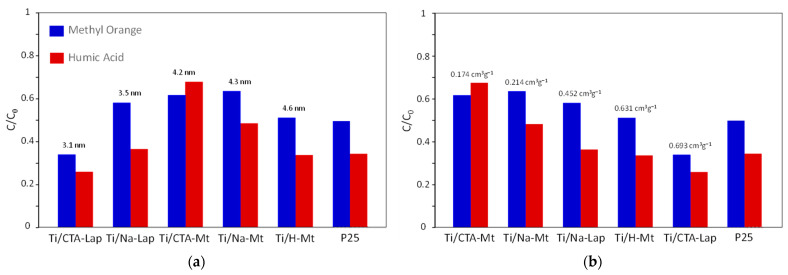
Results of photocatalytic experiments after 5 h irradiation: (**a**) composites ordered according to the increasing crystallinity of the titania component; (**b**) composites ordered according to the increasing pore volume.

All prepared composites proved to be active in the photocatalytic destruction of both substrates, but their performance differed. The results of the conducted photocatalytic experiments are presented in Figure 7a,b in two ways. In Figure 7a, the ordering of composites follows the increasing crystallinity of the titania component, while in Figure 7b, the photocatalysts are ordered according to the increasing pore volume. Both factors, i.e., the size of anatase crystallites and the development of textural parameters, have long been identified as factors critical for photocatalysis. Increased crystallinity provides better charge separation, which improves photoactivity by reducing the charge recombination effect. On the other hand, a well-developed porosity ensures good access to deposited titania particles and facilitates the diffusion of reactant molecules [45].

Analysis of the data shows that for both investigated photocatalytic reactions, no straightforward correlation with the TiO_2_ crystal size can be found (Figure 7a). Instead, a bell-shaped dependence is observed. This result can be explained by the mutual opposite influence of two factors. On the one hand, an increase in the size of TiO_2_ crystals provides better charge separation, which improves photoactivity by reducing the effect of charge recombination, but on the other hand, it means a decrease in the specific surface area of the titania component, which is unfavorable for photocatalysis. In contrast, both methyl orange and humic acid photodegradation show essentially monotonic increases in photocatalytic activity as a function of total pore volume in the composites. This shows that in the case of materials prepared in the present work, the photocatalytic efficiency is primarily controlled by the development of textural properties, i.e., by the access of reactants to photocatalytically active species at the surface of TiO_2_ NPs.

Noteworthy, the most photoactive composites, Ti/CTA-Lap and Ti/H-Mt, contain clay components whose structure was partially destroyed. In the case of Ti/H-Mt, the destruction occurred at the clay synthesis stage, resulting in increased porosity even before loading with TiO_2_ nanoparticles. In contrast, partial destruction of the CTA-Lap component occurred during composite synthesis as a result of interaction with the acidic aqueous cores of inverse micelles containing TiCl_4_ solution. It is likely that the formation of an amorphous silicate product, whose presence in Ti/CTA-Lap was inferred from FTIR analysis, contributed to the favorable modification of the composite texture.

The graphs also show that both in the photodegradation of methyl orange and the humic acid, the performance of the best composite photocatalysts is comparable to or better than that of the P25 standard, known for its exceptionally high photoactivity. Moreover, the weight of P25 used in the experiments was the same as that of the composite photocatalysts, in which TiO_2_ accounted for 59.8 to 71.1% of the catalyst mass, further indicating the higher photoactivity of titania present in the composites. This effect can be attributed primarily to the several times smaller size of the supported TiO_2_ nanocrystals (3.1–4.6 nm compared to about 20 nm in P25 [46]), resulting in a larger specific surface area of the photoactive material, as well as a lower tendency to agglomeration due to immobilization in the clay matrix. This shows that the adopted approach to design TiO_2_–clay composite photocatalysts is very promising, especially since there are a number of synthetic parameters that can be modified to further optimize the catalyst properties.

A literature search conducted to evaluate the present results in the context of previous studies shows that among several papers on the photodegradation of methyl orange by TiO_2_ composites with montmorillonite and/or laponite [47,48,49,50,51,52,53], only two reported performance better than the reference P25 [51,53]. In both cases, it was the preparation of the photocatalysts that had the greatest impact on the outcome of methyl orange destruction. A preparative procedure developed by Butman et al. [51] resulted in the formation of TiO_2_-pillared montmorillonite in which the TiO_2_ nanoparticles exhibited a high degree of crystallinity and consisted of a mixture of anatase and rutile phases, considered beneficial for photocatalysis. Han et al. [53] used the acid sol method to load TiO_2_ onto montmorillonite support and promoted further the photoactivity of material by doping titania with yttrium.

As for the photodegradation of humic acid, the use of TiO_2_/clay mineral composites for this purpose is, at present, a barely studied area. The only paper we could find on the subject is a recent report by Soison et al. [54], who developed an adsorbent based on a mixture of P25 and commercial kaolin clay and found that its ability to remove humic acid from simulated wastewater (92% in 12 h) was enhanced by UV radiation (100% in 8 h). Given the lack of other information on the photocatalytic degradation of humic acid by TiO_2_/clay systems, it is worth mentioning the work of Kim et al. [55], who studied a composite photocatalyst prepared from commercial nanosized ZnO and laponite. This material proved to be very active in the photodegradation of humic acid, achieving, under the experimental conditions used, 90% removal of the contaminant within 1 h of UV irradiation. Thus, the high photocatalytic activity of TiO_2_/laponite composites in destroying humic acid, found in the present study, is consistent with the above data and confirms the potential of laponite as a carrier of photoactive semiconductor nanoparticles.

## 3. Materials and Methods

### 3.1. Materials

The sodium form of montmorillonite (Mt), denoted Na-Mt, was obtained from the less than 2 µm particle size fraction of bentonite (Jelšový Potok, Slovakia) by cation exchange with excess of 1 M NaCl solution, followed by washing with distilled water until negative reaction of the solute with AgNO_3_. The cetyltrimethylammonium form of montmorillonite, denoted CTA-Mt, was prepared from Na-Mt by cation exchange with excess of cetyltrimethylammonium bromide (CTABr) aqueous solution, followed by washing with distilled water until negative reaction of the solute with AgNO_3_. CTA-Mt was subsequently washed with isopropanol and stored in the form of a wet paste. The acid form of montmorillonite, denoted H-Mt, was prepared as described in [56]. Briefly, Na-Mt, pretreated in a drying box at 200 °C, was subjected to dry impregnation with 6 M H_2_SO_4_, followed by washing with distilled water until a negative reaction of the solute with BaCl_2_. The sodium form of laponite, laponite RD, denoted Na-Lap, was purchased from BYK-Chemie GmbH (Wesel, Germany). The cetyltrimethylammonium form of laponite, denoted CTA-Lap, was prepared from Na-Lap by cation exchange with excess CTABr aqueous solution, followed by washing with distilled water until negative reaction of the solute with AgNO_3_. CTA-Lap was subsequently washed with isopropanol and stored in the form of a wet paste.

Ti-containing inverse microemulsion was synthesized according to the procedure described in detail in [40] (method C). In a typical experiment, aqueous Ti sol (prepared by controlled hydrolysis of TiCl_4_), CTABr, and 1-hexanol were mixed together in the weight ratio 17:28:55 and stirred until the mixture turned transparent.

Two methods of composite synthesis were used, differing in the manner of preparation of the clay mineral dispersion (as described in [26], with minor modifications). Briefly, exfoliated organoclays for composite synthesis (CTA-Mt and CTA-Lap) were prepared as suspensions in 1-hexanol by intense stirring for 48 h, while aqueous suspensions of inorganic clay forms (Na-Mt, H-Mt, and Na-Lap) stirred for 48 h were mixed with CTABr and 1-hexanol in weight proportion 17:28:55 until stable dispersion in the form of water-in-oil emulsion was obtained. Further synthesis stages were common for both organic and inorganic clay derivatives and consisted of mixing the clay dispersions with Ti-containing inverse microemulsion in the amount providing the intended TiO_2_ loading of 1.5 g per gram of clay. To accelerate Ti precursor hydrolysis, 10% NH_3_ aq was added dropwise until the suspensions turned white. The mixtures were then heated under constant stirring at 80 °C for 3 h. After cooling down, the suspensions were washed by centrifugation with ethanol (3×), followed by washing with a 1:1 water–ethanol mixture until free of bromide ions, and then dried in a drying box at 50 °C. Thus, prepared composites are further referred to as Ti/Na-Mt, Ti/CTA-Mt, Ti/H-Mt, Ti/Na-Lap, and Ti-CTA-Lap.

TiCl_4_ (ReagentPlus^®^) and 1-hexanol (for synthesis) were purchased from Sigma-Aldrich (Poznań, Poland); all other chemicals used in syntheses were of p.a. purity provided by Chempur (Piekary Slaskie, Poland). P25 TiO_2_ reference photocatalyst was manufactured by Evonik (Essen, Germany).

### 3.2. Methods

Powder X-ray diffraction (XRD) patterns were recorded using an X’Pert PRO MPD diffractometer (PANalytical, Almelo, The Netherlands) with CuKα radiation. The crystallite sizes of TiO_2_ nanoparticles (the size of coherently scattering domains) were estimated for the (101) reflection of anatase using the Scherrer formula and taking into account the instrumental broadening.

Scanning/transmission electron microscopy/energy dispersive X-ray spectroscopy (SEM/TEM/EDX) study of the materials was carried out with the aid of JEOL JSM-7500F Field Emission Scanning Electron Microscope (JEOL, Tokyo, Japan) coupled with an INCA PentaFetx3 EDX (Oxford Instruments, Abingdon, UK) system. SEM/TEM images were recorded for the uncoated samples deposited as suspensions on 200 mesh copper grids covered with carbon support film. An average of EDX measurements for 10 areas of ca. 1 μm × 1 μm, chosen at random on the sample surface, was used for determination of sample composition.

Transmission Fourier transform infrared (FTIR) spectra were recorded using a Nicolet 6700 (Thermo Scientific, Madison, WI, USA) spectrometer in the 4000–400 cm^−1^ range at a spectral resolution of 2 cm^−1^ for samples prepared as KBr discs.

A combined thermogravimetric (TG) and differential scanning calorimetry (DSC) analysis was carried out in the flow of air (40 mL/min) with an STA 409 PC LUXX TG/DSC apparatus (Netzsch, Selb, Germany) in the temperature range of 30–1000 °C and at a heating rate of 10 °C/min.

N_2_ adsorption/desorption at −196 °C was measured with an AUTOSORB 1 (Quantachrome, Boynton Beach, FL, USA) instrument. The samples were outgassed at 150 °C for 2 h. Brunauer–Emmett–Teller (BET) formalism was used for the calculation of specific surface areas (S_BET_). The total pore volume (V_tot_) was determined from the amount of N_2_ adsorbed at *p*/*p*_0_ = 0.996. Pore size distribution (PSD) profiles were determined from the adsorption branch using the non-local density functional theory (NL DFT) method. The mean pore diameter (D_av_) was calculated with the D_av_ = 4V_tot_/S_BET_ Gurvitch formula.

**Figure 8:**
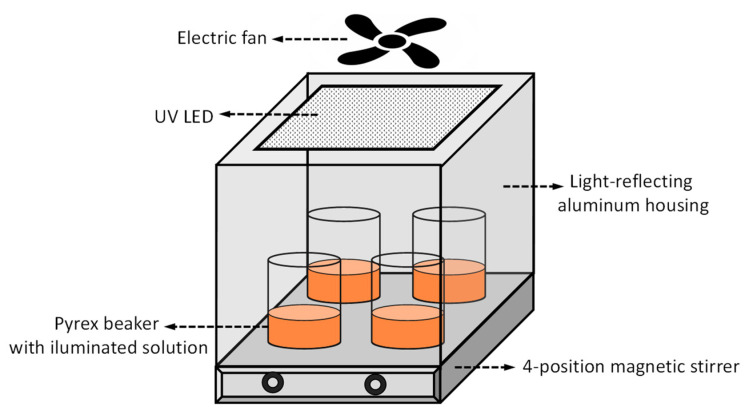
Scheme of setup for photocatalytic experiments.

The photocatalytic activity of the synthesized composites was evaluated in the decomposition of methyl orange (MO) (for microscopy, Sigma-Aldrich, Poznan, Poland) (used as 5 × 10^−5^ M aqueous solution) and humic acid (HA) (Na salt, technical grade, Sigma-Aldrich, Poznan, Poland) (used as 40 mgL^−1^ aqueous solution, after filtering to remove coarse particles). The scheme of experimental setup is shown in Figure 8. The reaction was carried out at room temperature in beakers made of Pyrex glass transparent to the UV radiation used in the experiments, placed on a 4-position magnetic stirrer, and illuminated from the top by a 365 nm light-emitting diode (LED) 50 W lamp (EcoEnergy, Słupsk, Poland). The illuminated area was about 20 cm^2^, and its distance from the radiation source was 25 cm. To make sure that all four positions on the magnetic stirrer plate were equivalent to each other, a series of measurements with identical parameters (except for position) were performed. In comparative measurements, such a configuration helps eliminate random experimental errors. The whole setup was surrounded by a light-reflecting aluminum housing. To avoid raising the temperature inside the housing, the reactor was cooled by an electric fan. Considering that, from a practical point of view, what matters is the mass of the catalyst that needs to be used to achieve the expected results, we decided to compare the activity of photocatalysts with identical process parameters, including the mass of the catalyst. In a typical experiment, a 0.04 g photocatalyst was placed in a 50 mL aqueous solution of MO or HA and stirred for 1 h in the dark to ensure the adsorption–desorption equilibrium before illumination. Subsequently, the UV irradiation was carried out for 5 h. The concentration of a pollutant was determined by taking a 3 mL sample of the reaction mixture, centrifuging it, and measuring light absorption at 465 nm (for MO) or 254 nm (for HA) using Lambda 35 Perkin Elmer UV–Vis spectrometer (PerkinElmer, Waltham, MA, USA). To evaluate the change in pollutant concentration due to water evaporation, the solutions were weighed before and after the experiment, and the necessary correction was introduced into the calculations. The photodegradation of pollutants is presented in terms of C/C_0_, where C_0_ is the absorbance of the pollutant solution before irradiation, and C is the absorbance at the end of the irradiation experiment.

## 4. Conclusions

A novel synthetic approach involving the deposition of TiO_2_ nanoparticles prepared by inverse microemulsion on exfoliated clay minerals was used to produce a series of composites with five different clay mineral components: Na-, CTA-, and H-form of montmorillonite and Na- or CTA-form of laponite. Physico-chemical characterization revealed that the synthesis environment strongly influenced the structure and composition of clay components. In the case of montmorillonites, the interlayer became filled by a mixture of CTA^+^ and hydronium ions, irrespective of the nature of the parent clay. The main effect in the case of the laponite component was the partial destruction of the silicate structure due to interaction with the acidic aqueous cores of the TiCl_4_-bearing inverse micelles. TiO_2_ formed anatase nanoparticles with a diameter of 4–6 nm and crystallinity depending on the type of clay component. The composites were characterized by high specific surface area and uniform mesoporosity, determined by the size of TiO_2_ nanoparticles. The best textural parameters were shown by composites containing clay components whose structure was partially destroyed, either during composite synthesis (laponite-based samples) or at the stage of clay preparation (acid-treated H-form of montmorillonite).

All synthesized materials were active in the photodegradation of model water pollutants, methyl orange and humic acid, but their performance differed. As the TiO_2_ crystal size increased, the photoactivity showed a bell-shaped dependence as a result of the mutual opposing influence of two factors: increasing charge separation, beneficial for photocatalysis, and decreasing the specific surface area of the titania component, unfavorable to the process. On the other hand, a direct correlation was found with the textural parameter, as the activity of the composites increased with increasing pore volume. The result showed that the photocatalytic performance was mainly controlled by the development of textural properties, which facilitate the transport of reactants to the active sites at the surface of immobilized TiO_2_ NPs. In both reactions, the performance of the most photoactive composites was better than that of the reference P25, proving the attractiveness of the proposed approach to photocatalyst design.

## Figures and Tables

**Table 1:** Chemical composition of investigated materials (based on EDX analysis) and textural parameters from N_2_ adsorption/desorption isotherms at −196 °C.

Sample	TiO_2_ [wt. %]	SiO_2_ [wt. %]	Al_2_O_3_ [wt. %]	MgO [wt. %]	Fe_2_O_3_ [wt. %]	Na_2_O [wt. %]	S_BET_ [m^2^g^−1^]	V_tot_ [cm^3^g^−1^]	D_av_ [nm]	D_dom_ [nm]	Anatase Cryst. Size [nm]
Ti/Na-Mt	61.8	26.2	9.7	1.4	0.9	-	181	0.214	4.7	4.9	4.3
Ti/CTA-Mt	60.7	26.8	9.9	1.5	1.1	-	154	0.172	4.5	4.6	4.2
Ti/H-Mt	59.8	28.5	9.1	1.5	1.1	-	350	0.631	7.2	5.7	4.6
Ti/Na-Lap	64.0	26.6	-	9.4	-	-	415	0.452	4.4	5.3	3.5
Ti/CTA-Lap	71.1	21.8	-	7.1	-	-	420	0.693	6.7	6.6	3.1
Na-Mt	-	65.6	24.9	4.5	2.1	2.9	41	0.089	4.3	3.7	-
CTA-Mt	-	66.5	25.8	4.9	2.8	-	10	0.071	29.4	6.4	-
H-Mt	-	70.6	22.8	3.9	2.7	-	181	0.435	9.7	3.4	-
Na-Lap	-	66.5	-	30.5	-	3.0	365	0.293	3.1	4.3	-
CTA-Lap	-	68.9	-	31.1	-	-	213	0.227	4.3	3.5	-

S_BET_—BET specific surface area, V_tot_—total pore volume, D_av_—average pore dimension, D_dom_—dominant pore size.

## Data Availability

The data presented in this study are available on request from the authors.
